# Serum Exosomes MicroRNAs Are Novel Non-Invasive Biomarkers of Intrahepatic Cholestasis of Pregnancy

**DOI:** 10.3389/fendo.2022.832577

**Published:** 2022-05-04

**Authors:** Ruirui Dong, Ningzhen Ye, Jing Wang, Shaojie Zhao, Tiejun Wang, Gaoying Wang, Xinrui Shi, Jing Cheng, Yan Zhang, Tingting Yao, Minjian Chen, Ting Zhang, Liang Luo

**Affiliations:** ^1^ The Affiliated Wuxi Maternity and Child Health Care Hospital of Nanjing Medical University, Wuxi, China; ^2^ School of Medicine, Jiangnan University, Wuxi, China; ^3^ State Key Laboratory of Reproductive Medicine, Institute of Toxicology, School of Public Health, Nanjing Medical University, Nanjing, China; ^4^ Key Laboratory of Modern Toxicology of Ministry of Education, School of Public Health, Nanjing Medical University, Nanjing, China; ^5^ Intensive Care Medicine, The Affiliated Wuxi No. 2 People’s Hospital of Nanjing Medical University, Wuxi, China

**Keywords:** intrahepatic cholestasis of pregnancy (ICP), exosomes, microRNA, biomarker, lipid metabolism, apoptosis

## Abstract

**Background:**

Intrahepatic cholestasis of pregnancy (ICP) is closely related to the occurrence of adverse outcomes. Currently, total bile acids (TBAs) are the only diagnostic index for ICP, and its sensitivity and specificity have certain limitations. In this study, we aimed to develop potential biomarkers for the diagnosis of ICP.

**Methods:**

Sixty pregnant women diagnosed with ICP and 48 healthy pregnant controls were enrolled in this study. We used the Agilent microRNA (miRNA) array followed by quantitative reverse transcriptase polymerase chain reaction assays to identify and validate the serum exosome miRNA profiles in ICP and healthy pregnant controls. We employed bioinformatics to identify metabolic processes associated with differentially expressed serum exosome miRNAs.

**Results:**

The expression levels of hsa-miR-4271, hsa-miR-1275, and hsa-miR-6891-5p in maternal serum exosomes were significantly lower in ICP patients compared to controls; the diagnostic accuracy of hsa-miR-4271, hsa-miR-1275, and hsa-miR-6891-5p was evaluated with the area under the receiver operating characteristic curve (AUC) values of 0.861, 0.886, and 0.838, respectively. Multiple logistic regression analysis showed that a combination of the levels of hsa-miR-4271and hsa-miR-1275 afforded a significantly higher AUC (0.982). The non-error rate of a combination of all three exosome miRNAs was the highest (95%), thus more reliable ICP diagnosis. The expression levels of all three exosome miRNAs were negatively associated with TBAs. Furthermore, according to bioinformatics analysis, the three exosome miRNAs were related to lipid metabolism, apoptosis, oxidative stress, and the Mitogen Activated Protein Kinase (MAPK) signaling pathway.

**Conclusions:**

This study may identify the novel non-invasive biomarkers for ICP and provided new insights into the important role of the exosome miRNA regulation in ICP.

## Introduction

Intrahepatic cholestasis of pregnancy (ICP) is the most common pregnancy-specific liver disease. The incidence rate is about 0.1%–2% (1), it varies in different regions and races (2). ICP is rarely harmful to the mother, except for an aggravating pruritus (3). The symptom will be relieved after birth within 48 h (4). However, ICP is associated with the increased risk of fetal disease such as staining of amniotic fluid, spontaneous preterm labor, fetal growth restriction (FGR), and even stillbirth (5). The mechanisms responsible for the development of ICP are still not fully understood. Total bile acid (TBA) is the final product of cholesterol catabolism in the liver and is closely related to the absorption, metabolism, and regulation of cholesterol. Today, the diagnosis of ICP is the increase level of TBA combination with typical pruritus. However, the specificity and the sensitivity of TBA are limited (6). Sentilhes et al. (7) have reported a case of a patient with ICP with low bile acid values (<13 UI/L) where fetal death occurred at 39.4 weeks, and the diagnostic accuracy of TBA might have been overestimated. Therefore, novel molecular biomarkers are urgently needed to improve diagnosis and prognosis of ICP.

MicroRNAs (miRNAs) are small noncoding RNA molecules. MiRNAs bind to the 3′ untranslated region (3′UTR) of the target messenger RNA (mRNA) (8), leading to the repression of protein translation or mRNA degradation. In our previous study, we have explored the differential miRNA expression profiles in ICP serum (9). Hence, miRNAs have potential as diagnostics biomarkers and therapeutic targets for ICP. However, miRNAs cannot stay in serum stably because of RNase enzyme. Exosomes are about 40~150 nm in size, which are cell-derived membrane vesicles (10). Different bioactive molecules such as proteins, DNAs, and RNAs are contained in exosomes. Those molecules play important roles in intercellular communication. Because of the protection of phospholipid (PL) bimolecular layer, the miRNAs contained in it cannot be easily degraded (11). However, few reports are found to focus on the diagnostic values of serum exosome miRNAs as biomarkers for ICP.

In this study, we used the Agilent miRNA array to explore the serum exosome miRNA profiles. The objective of this study was to elucidate ICP-specific or abnormally expressed serum exosome miRNAs that could serve as biomarkers for the diagnosis of ICP. Finally, for our future research, we explore their targeted gene through the gene ontology (GO) and Kyoto Encyclopedia of Genes and Genomes (KEGG) pathway, which provides new ideas to explain potential molecular mechanism of ICP.

## Materials and Methods

### Patients and Samples

Sixty pregnant women diagnosed with ICP and 48 healthy pregnant controls were selected. The gestational week of the participants was the third trimester (>28 weeks). They all received care at the Affiliated Wuxi Maternity and Child Health Care Hospital of Nanjing Medical University from September 2020 to June 2021, and none of the participants were infected with SARS-COV-2. We selected three patients with ICP and three healthy pregnant controls for preliminary miRNA array analysis, and the serum sample size was also 3 in each group. At the same time, 60 and 48 patients were selected for validation of serum exosome miRNAs in ICP and control group, respectively, and the three patients used to screen for differential miRNAs were also used for validation. Because of the large amount of serum required for exosome extraction, the serum of every three patients was mixed for exosome extraction. The serum sample size was 20 in ICP group and 16 in control group. All real-time amplifications were carried out in triplicate *via* an ABI 7500 Real-Time PCR system (Applied Biosystems).

All samples were from primiparous Chinese women with singleton pregnancies. Women presenting with classical pruritus associated with raised serum bile acids were diagnosed with ICP. All patients subsequently resolved after delivery. The diagnostic criteria of ICP as well as both inclusion and exclusion criteria have previously been described (12). Briefly, patients suffering from other diseases, which could cause the rise of serum bile acids, were excluded from analysis, such as viral hepatitis; preeclampsia; hemolysis, elevated liver enzymes, and low platelets (HELLP) syndrome; primary biliary cirrhosis; acute fatty liver of pregnancy; and any ultrasound abnormalities that could have resulted in biliary obstruction. Venous blood samples were collected from all participants and separated within 4°C by centrifugation at 4,000 rpm for 10 min. The amount of serum required for extraction of exosomes in the Agilent miRNA array and quantitative reverse transcriptase polymerase chain reaction (qRT-PCR) assay was 4 ml. The obtained serum was then centrifuged for 10 min at 5,000 g and for 10 min at 3,000 g to remove cells and cell debris. Each samples were stored at −80°C until to analyses. No patient underwent ursodeoxycholic acid treatment prior to blood sample collection. Ethics approval was granted by the Institutional Review Board of Nanjing Medical University, and all participants provided written, informed consent [Nanjing Medical University Ethical Review (2016), number 241].

### Exosomes Isolation and Identification

Exosomes were extracted using ExoQuick solution (EXOQ5A-1; SBI System Biosciences, USA) according to the manufacturer’s instructions. Briefly, 120 μl of ExoQuick solution was added at 500 μl of serum, and the sample was mixed and incubated at 4°C for 30 min, followed by centrifugation for 30 min at 1,500 g. Finally, exosomes were obtained after pellets in the bottom of the tube were resuspended with 400 μl of phosphate-buffered saline (PBS). Exosomes were stored at −80°C for characterization and RNA isolation.

The characteristics of isolated exosomes were examined by transmission electron microscopy (TEM). Briefly, exosome pellets were resuspended in PBS and loaded onto a copper grid with supporting film, fixed at least 5 min, and remove excess fluid. Then, the exosomes were stained with 3% phosphate tungsten for 3 min. After being air-dried, the grids were visualized with TEM at 80 kV. The size distribution and concentration of exosomes was analyzed by nanoparticle tracking analysis (NTA) and its corresponding software. Exosomes were diluted with PBS, and 100 μl of the sample was loaded into the exosome analysis chamber. We then collected dynamic images and analyzed the concentration and distribution of the exosomes. To verify the isolation of exosomes from the serum, Western blot (WB) were used to analyze CD9, CD63, and TSG101. Total proteins were lysed with Radio-Immunoprecipitation Assay (RIPA) and separated by 10% Sodium Dodecyl Sulfate Polyacrylamide Gel Electrophoresis (SDS-PAGE) gels and then transferred to polyvinylidene fluoride membranes. After blocking with 5% non-fat milk for 1 h, the membranes were incubated with primary antibody at 4°C overnight. Then, the membranes were incubated with the secondary antibody anti-rabbit Immunoglobulin G (IgG) for 1 h at 37°C. Finally, the protein bands were visualized by chemiluminescence using SuperSignal West Pico.

### The miRNA Array

The miRNA array was performed as previously described in (9). Briefly, we used the TRIzol reagent (Invitrogen) and purified with the mirVana miRNA Isolation Kit (Ambion, Austin, TX, USA) to extract total RNAs containing small RNAs from serum exosome samples according to the manufacturer’s protocol. The miRNA profiling was performed using Agilent miRNA array. The Agilent miRNA array, which features eight identical subarrays per slide (in 8 × 60K format); each subarray contained probes interrogating 2,549 human mature miRNAs derived from miRBase version R21.0. Each miRNA was probed 30 times. The array also contains 2,164 Agilent control probes. All microarray experiments were performed as instructed by the manufacturer. Briefly, the miRNAs were labeled using the Agilent miRNA labeling reagent. Total RNA (200 ng) was dephosphorylated and ligated with pCp-Cy3; the labeled RNA was purified and hybridized to miRNA arrays. Images were scanned using the Agilent microarray scanner (Agilent) and gridded, and the data were analyzed using Agilent feature extraction software version 10.10.

The miRNA array data were analyzed for data summarization, normalization, and quality control by using the GeneSpring software V13 (Agilent). The default 90th percentile normalization method was performed for date preprocessing. When selecting differentially expressed miRNAs, we used threshold values of ≥1.5- and ≤0.67-fold change, and P < 0.05 was considered statistically significant. The data were log2-transformed and median-centered by genes using the Adjust Data function of CLUSTER 3.0 software and then further analyzed with hierarchical clustering with average linkage (13). Finally, we used Java TreeView (Stanford University School of Medicine, Stanford, CA, USA) to perform tree visualization.

### Bioinformatics Analyses

The GO biological processes (BPs), cellular component (CC), and molecular functional (MF) annotations of genes targeted by all differentially expressed miRNAs were analyzed. The KEGG pathway was used to analyze their associated signal pathway of differentially expressed miRNAs. The target gene of the three miRNAs in ICP was performed using the cytoscape software.

### Validation of Differential Serum Exosome MiRNAs Expression

Total RNAs was reversed transcribed using a PrimeScript™ RT Reagent Kit (Takara, China). The TaqMan microRNA assay (Applied Biosystems, Inc.) was employed to quantify relative miRNA expression levels using miR-16 as the endogenous control. The primer sequences used to amplify the miRNAs are summarized in **Table 1**. In brief, 100 ng of RNA was used as a template and then reverse-transcribed using a miRNA-Specific Stem-Loop RT Primer. The universal reverse primer and a specific forward primer were used to further amplify the resulting cDNA. The cycle parameters were carried out under the following condition: 95°C for 10 min followed by 40 cycles of 95°C for 15 s and 60°C for 1 min. Because of the large amount of serum required for exosome extraction, the serum of every three patients was mixed for exosome extraction. All real-time amplifications were carried out in triplicate *via* an ABI 7500 Real-Time PCR system (Applied Biosystems). The fold changes in miRNA levels were calculated using the 2^−ΔΔCT^ method.

**Table 1:** Primers used in this study.

Gene Name		Primer Sequence 5′→3′
hsa-miR-4271	RT	GTCGTATCCAGTGCAGGGTCCGAGGTATTCGCACTGGATACGACgacagc
	AS	GTGGGGGAGAGGCTGTC
hsa-miR-1275	RT	GTCGTATCCAGTGCAGGGTCCGAGGTATTCGCACTGGATACGACgggaga
	AS	TCGCCTCCTCCTCTCC
hsa-miR-6891-5p	RT	GTCGTATCCAGTGCAGGGTCCGAGGTATTCGCACTGGATACGACcccctc
	AS	ATAAGGAGGGGGATGAGG
hsa-miR-16	RT	GTCGTATCCAGTGCAGGGTCCGAGGTATTCGCACTGGATACGACaaaata
	AS	TGAAGCGTTCCATATTTTGTC

### Statistical Analysis

All data were analysis with SPSS v. 22.0 (IBM, Armonk, NY, USA) and GraphPad Prism software. The data were normally distributed. Student’s t-test was used to compare the differences in clinical features and biomarkers between ICP and control groups. We used receiver operator characteristic (ROC) curve analysis to assess the diagnostic value of biomarkers for ICP and derive sensitivities and specificities [with 95% confidence intervals (CIs)] by referencing areas under the curves (AUCs); an AUC greater than 0.70 was considered an acceptable level of discrimination. Pearson correlations between differentially expressed serum exosome miRNA levels and serum TBA levels were also calculated. ROC curve of a combination of two or more miRNAs was derived using multiple logistic regression (MLR) analyses. All results were expressed as means ± standard errors (SEs). A P-value <0.05 was considered to reflect statistical significance.

## Results

### Clinical Characteristics of Pregnant Women with ICP and Healthy Pregnant Controls

The basic clinical information and perinatal outcomes of 60 pregnant women with ICP and 48 controls are summarized in **Table 2**. As for the maternal age, gestational weeks at the time of blood collection and newborn weight, we found no significant difference among the two groups (both P > 0.05). However, the levels of TBA, alanine transaminase (ALT), and aspartate transaminase (AST) were observably higher in pregnant women with ICP than in healthy pregnant controls (**Table 2**). Gestational weeks at the time of delivery were observably lower in pregnant women with ICP than in healthy pregnant controls (**Table 2**).

**Table 2:** Clinical characteristics of pregnant women with ICP and healthy pregnant controls.

Variable	Screening Samples			Validation Samples		
	ICP (n = 3)	Control (n = 3)	P value	ICP (n = 60)	Control (n = 48)	P value
Maternal age (years)	29.3 ± 1.5	29.7 ± 0.6	0.742	29.4 ± 2.7	28.9 ± 2.8	0.302
Blood collection weeks	36.2 ± 1.7	35.3 ± 1.1	0.463	36.4 ± 1.8	36.7 ± 1.5	0.857
TBA (μmol/L)	93.8 ± 49.2	3.0 ± 0.2	0.033*	37.7 ± 32.9	3.0 ± 1.3	0.000*
ALT (IU/L)	286.2 ± 143.2	16.6 ± 9.5	0.031*	61.5 ± 114.3	10.8 ± 5.4	0.002*
AST (IU/L)	180.5 ± 106.4	20.1 ± 8.2	0.060	47.6 ± 66.1	16.9 ± 5.1	0.001*
Delivery weeks	36.6 ± 1.9	40.4 ± 0.3	0.025*	37.9 ± 1.7	39.3 ± 1.3	0.000*
Newborn weight (g)	2763.3 ± 257.4	3643.3 ± 476.1	0.065	3112.0 ± 601.1	3271.3 ± 398.1	0.102

TBA, total bile acid; ALT, alanine transaminase; AST, aspartate transaminase. Statistical analyses were performed using the t-test. All results were expressed as means ± standard errors (SEs); *P < 0.05 was considered to reflect significance.

### Characterization of Serum Exosomes

**Figure 1:**
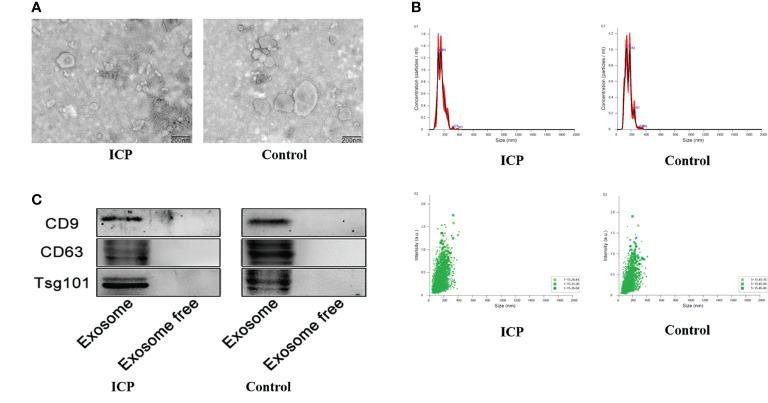
Characterization of serum exosomes extraction. **(A)** Transmission electron microscopy showing the spherical shape of exosomes. **(B)** Nanoparticle tracking analysis showing the size distribution and concentration of exosomes. **(C)** Western blot showing the expression of exosomes protein markers including CD9, CD63, and TSG101.

The exosomes were identified through TEM analysis that revealed that the small vesicles with diameters ranging from approximately 50 to 150 nm and a lipid bilayer could be observed under the TEM (**Figures 1A, B**). The exosomes were identified through WB analysis revealed the expression of CD9, CD63, and TSG101 (**Figure 1C**), which showed specific bands in exosomes pellets but not in exosome-depleted supernatant (EDS). From this evidence, we demonstrated the existence of exosomes in serum.

### Screening and Identification of Differentially Exosome miRNAs

**Figure 2:**
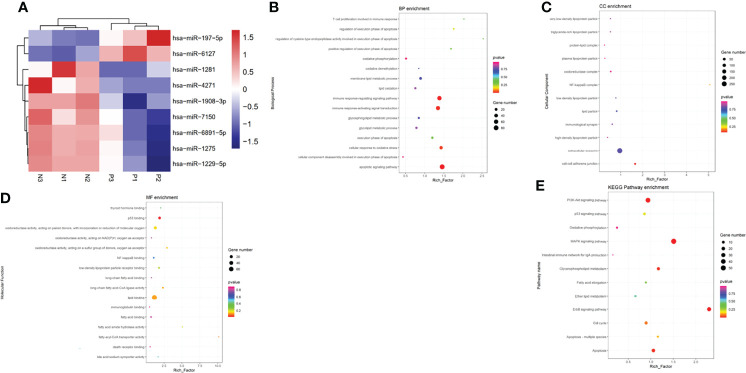
Bioinformatics analysis of differentially expressed serum exosome miRNAs. **(A)** Cluster analyses of differentially expressed miRNAs in the serum exosome of pregnant women with ICP (P) and healthy pregnant women (N). Hierarchical cluster analyses of the nine differentially expressed miRNAs displaying significantly altered expression levels in patients with ICP. Expression levels are shown as colored boxes; red, high level; blue, low level. **(B)** GO classifications and enrichments in terms of BP. **(C)** GO classifications and enrichments in terms of CC. **(D)** GO classifications and enrichments in terms of MF. **(E)** KEGG pathway enrichment analyses.

Ultimately, a total of 183 miRNAs in serum exosome of patients with ICP were screened according to the miRNA array. The criteria for differential expression were 1.5-fold upregulation or 0.67-fold downregulation and a Benjamin-Hochberg corrected P-value of 0.05. A total of nine dysregulated miRNAs meeting these criteria were selected as candidates for diagnostic markers. Among the different miRNAs, two exhibited a remarkable increase in relative intensities, whereas seven were found to be decreased in serum exosome of patients with ICP compared with controls (**Table 3**). Cluster analyses were used to define specific expression patterns of all nine miRNAs; expression levels significantly differed among pregnant women with ICP and healthy pregnant controls (**Figure 2A**).

**Table 3:** Differentially expressed serum exosome miRNAs among pregnant women with ICP and healthy pregnant women.

Exosome miRNA	Fold Change (P:C)	P-value	Change in Expression Level
hsa-miR-197-5p	1.54	0.036	UP
hsa-miR-6127	1.59	0.004	UP
**hsa-miR-4271**	**0.50**	**0.027**	**DOWN**
**hsa-miR-1275**	**0.33**	**0.031**	**DOWN**
hsa-miR-1229-5p	0.65	0.036	DOWN
hsa-miR-7150	0.45	0.031	DOWN
hsa-miR-1908-3p	0.47	0.011	DOWN
hsa-miR-1281	0.49	0.029	DOWN
**hsa-miR-6891-5p**	**0.45**	**0.044**	**DOWN**

The miRNA array data were analyzed for data summarization, normalization, and quality control by using the GeneSpring software V13 (Agilent). The default 90th percentile normalization method was performed for date preprocessing. The table contains quantitative data for miRNAs that were ≥1.5-fold upregulated or ≤0.67-fold downregulated, and P < 0.05 was considered statistically significant in pregnant women with ICP (P) compared to healthy pregnant women (C). The P:C ratios are shown. The key miRNAs data (verified via qRT-PCR) are bolded.

### Gene Ontology Enrichment and Pathway Enrichment Analyses of Differentially Expressed MiRNAs

We determined the GO enrichments associated with differentially expressed miRNAs. The miRNAs were involved in many BPs including the lipid oxidation, membrane lipid metabolic process, immune response-regulating signaling pathway, cellular response to oxidative stress, and apoptotic signaling pathway (**Figure 2B**). The miRNAs were also involved in many CC activities including the oxidoreductase complex, immunological synapse, extracellular exosome, lipid particle, NF-kappa B complex, and cell-cell adherens junction (**Figure 2C**). The miRNAs were involved in many aspects of MFs including the P53 binding, fatty-acyl-CoA transporter activity, lipid binding, oxidoreductase activity, thyroid hormone binding, bile acid:sodium symporter activity, death receptor binding, and immunoglobulin binding (**Figure 2D**). KEGG pathway analyses indicating that the differentially expressed miRNAs were involved in fatty acid elongation, P53 signaling pathway, MAPK signaling pathway, ErbB signaling pathway, PI3K-Akt signaling pathway, apoptosis, intestinal immune network for IgA production, cell cycle, and oxidative phosphorylation (**Figure 2E**).

**Figure 3:**
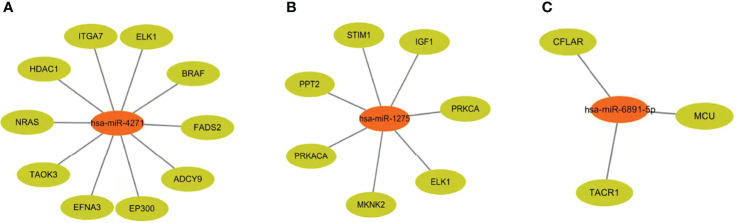
Target genes of differentially expressed serum exosome miRNAs. **(A)** Target genes associated with hsa-miR-4271. **(B)** Target genes associated with hsa-miR-1275. **(C)** Target genes associated with hsa-miR-6891-5p.

At the same time, we predicted the target genes of these expressed exosome miRNAs (hsa-miR-4271, hsa-miR-1275, and hsa-miR-6891-5p). Target genes associated with hsa-miR-4271 include ITGA7, ELK1, and BRAF (**Figure 3A**). Target genes associated with hsa-miR-1275 include STIM1, PPT2, and IGF1 (**Figure 3B**). Target genes associated with hsa-miR-6891-5p include CFLAR, MCU, and TACR1. (**Figure 3C**).

### Validation of Differentially Expressed Exosome MiRNAs

**Figure 4:**
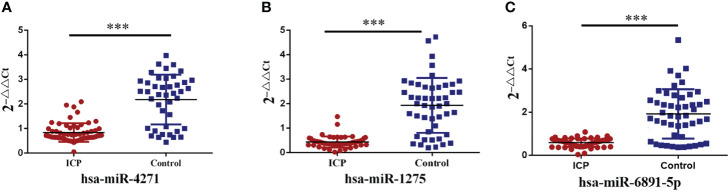
Validation of alteration in serum exosome miRNA levels. **(A)** Serum exosome levels of hsa-miR-4271 in ICP and control groups (P = 0.000). **(B)** Serum exosome levels of hsa-miR-1275 in ICP and control groups (P = 0.000). **(C)** Serum exosome levels of hsa-miR-6891-5p in ICP and control groups (P = 0.000); (***P <0.001).

Three differentially expressed exosome miRNAs (hsa-miR-4271, hsa-miR-1275, and hsa-miR-6891-5p) were determined by qRT-PCR assays. The hsa-miR-16 served as the normalization control. After filtering these miRNAs, we found that hsa-miR-4271, has-miR-1275, and has-miR-6891-5p were significantly downregulated in pregnant women with ICP compared with normal samples (both P < 0.001; **Figure 4**).

### Diagnostic Utility of Serum Differential Exosome MiRNA Levels

**Figure 5:**
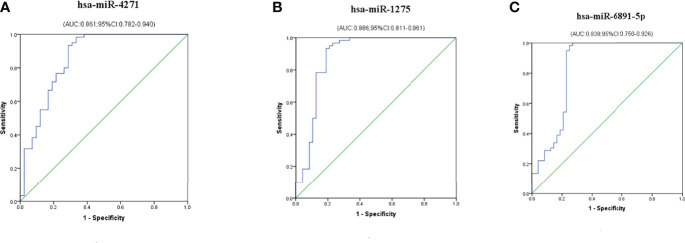
Diagnostic utility of single exosome miRNA levels in maternal serum in pregnant women with ICP. **(A)** hsa-miR-4271; **(B)** hsa-miR-1275; **(C)** hsa-miR-6891-5p.

To evaluate the probability of diagnosed with ICP, ROC curves were constructed and AUCs were calculated. The AUCs for hsa-miR-4271, hsa-miR-1275, and hsa-miR-6891-5p were 0.861, 0.886, and 0.838, respectively (**Figure 5**). We assessed the diagnostic utility of hsa-miR-4271, hsa-miR-1275, and hsa-miR-6891-5p at cutoff values of 1.51, 0.70, and 0.92, respectively; the Youden index confirmed that these cutoffs were optimal. The non-error rate, sensitivities, and specificities of hsa-miR-4271, hsa-miR-1275, and hsa-miR-6891-5p were 84.3%, 93.3%, and 71.4%; 87.0%, 96.7%, and 75.0%; and 86.9%, 98.3%, and 72.9%, respectively (**Table 4**).

**Table 4:** Diagnostic value analysis of serum exosome miRNAs in ICP.

Exosome MiRNA	Non-Error Rate	Sensitivity	Specificity	Cutoff Value (2^−ΔΔCT^)
hsa-miR-4271	84.3%	93.3%	71.4%	1.51
hsa-miR-1275	87.0%	96.7%	75.0%	0.70
hsa-miR-6891-5p	86.9%	98.3%	72.9%	0.92
hsa-miR-1275/6891-5p	88.8%	98.3%	77.1%	/
hsa-miR-4271/6891-5p	94.1%	96.6%	90.5%	/
hsa-miR-4271/1275	94.1%	96.7%	90.5%	/
hsa-miR-4271/1275/6891-5p	95.0%	96.6%	92.9%	/

ROC curve analysis to assess the diagnostic value of biomarkers for ICP and derive non-error rate, sensitivities, and specificities [with 95% confidence intervals (CIs)] by referencing AUCs; ROC curve of a combination of two or more miRNAs was derived using multiple logistic regression (MLR) analyses.

**Figure 6:**
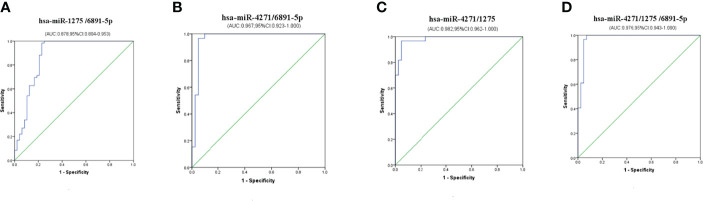
Diagnostic utility of combined exosome miRNA levels in maternal serum in pregnant women with ICP. **(A)** hsa-miR-1275/6891-5p; **(B)** hsa-miR-4271/6891-5p; **(C)** hsa-miR-4271/1275; **(D)** hsa-miR-4271/1275/6891-5p.

Multiple logistic regression analyses of combined biomarker data yielded an AUC of 0.878, 0.967, 0.982, and 0.976 for hsa-miR-1275/6891-5p, hsa-miR-4271/6891-5p, hsa-miR-4271/1275, and hsa-miR-4271/1275/6891-5p, respectively (**Figure 6**). The non-error rate, sensitivities, and specificities of hsa-miR-1275/6891-5p, hsa-miR-4271/6891-5p, hsa-miR-4271/1275, and hsa-miR-4271/1275/6891-5p were 88.8%, 98.3%, and 77.1%; 94.1%, 96.6%, and 90.5%; 94.1%, 96.7%, and 90.5%; and 95.0%, 96.6%, and 92.9%, respectively (**Table 4**). Thus, this was the solid evidence that the combination of these miRNAs could be reliable, and the novel biomarker improved the diagnostic value of the compounds.

### Correlation Analysis of Serum Differential Exosome MiRNAs With TBA and Gestational Week (Delivery)

**Figure 7:**
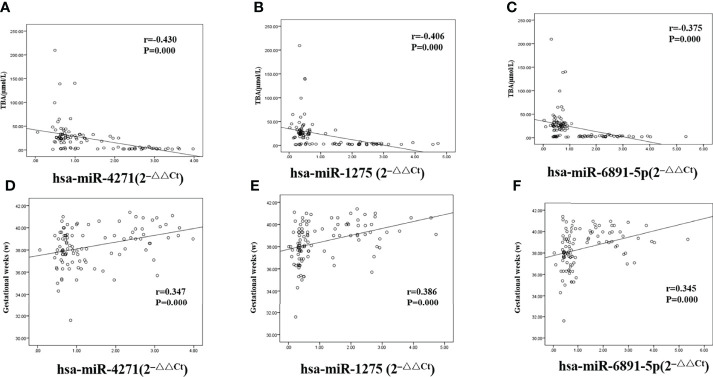
Correlations between serum levels of exosome miRNA and levels of total bile acid and gestational week (delivery) in pregnant women with ICP. **(A)** hsa-miR-4271 and TBA (r = −0.430 and P = 0.000); **(B)** hsa-miR-1275 and TBA (r = −0.406 and P = 0.000); **(C)** hsa-miR-6891-5p and TBA (r = −0.375 and P = 0.000); **(D)** hsa-miR-4271 and gestational weeks (r = 0.347 and P = 0.000); **(E)** hsa-miR-1275 and gestational weeks (r = 0.386 and P = 0.000); **(F)** hsa-miR-6891-5p and gestational weeks (r = 0.345 and P = 0.000).

We calculated Pearson correlations to find the relationship between levels of TBA and hsa-miR-4271, hsa-miR-1275, and hsa-miR-6891-5p; the levels of all three exosome miRNAs were found to be negatively associated with TBA levels (r = −0.430 and P = 0.000, r = −0.406 and P = 0.000, and r = −0.375 and P = 0.000 for hsa-miR-4271, hsa-miR-1275, and hsa-miR-6891-5p, respectively; **Figures 7A**–**C**). At the same time, we calculated Pearson correlations between gestational week (delivery) and hsa-miR-4271, hsa-miR-1275, and hsa-miR-6891-5p; the levels of all three exosome miRNAs were found to be positively associated with gestational week (delivery) (r = 0.347 and P = 0.000, r = 0.386 and P = 0.000, and r = 0.345 and P = 0.000 for hsa-miR-4271, hsa-miR-1275, and hsa-miR-6891-5p, respectively; **Figures 7D**–**F**).

## Discussion

ICP is associated with upset pregnancy outcomes, including spontaneous preterm labor, staining of amniotic fluid, and sudden intrauterine death. In this study, we found that the gestational week (delivery) of pregnant women with ICP was significantly lower than that of healthy pregnant controls, indicating an increased probability of preterm birth in patients with ICP. Therefore, early diagnosis and prediction of patients with ICP have indispensable clinical value. Unfortunately, current laboratory criteria for clinical diagnosis of ICP mainly rely on elevated levels of TBA, and their sensitivity and specificity are limited. Martinefski et al. (14) have reported that the patients with other hepatobiliary diseases including viral hepatitis, hepatolithiasis, autoimmune hepatitis, and acute fatty liver disease during pregnancy may also have high level of TBA. Manzotti et al. (15) searched 5,073 references and found that the sensitivity and specificity of TBA ranged from 0.72 to 0.98 and 0.81 to 0.97, respectively. Therefore, it is thus necessary to investigate potential early sensitive molecular events in the setting of ICP and screen for sensitive and specific biomarkers.

MiRNAs play an important role in biological function and pathology of disease. The miRNAs can be remained in a highly stable state because of the lipid bilayer membrane in exosome. Therefore, exosome miRNA has been widely used in mechanistic studies and to serve as potential clinical biomarkers of gynecological and pregnancy-related conditions. Zhang et al. (16) identified 157 differentially expressed exosome miRNAs in the placental tissue obtained from gestational diabetes mellitus (GDM) and found that exosome miRNA-125b and miRNA-144 can be excellent diagnostic value for GDM. Li et al. (17) found that seven miRNAs were differentially expressed in plasma exosomes from women with preeclampsia, which could provide insight into the pathophysiology of preeclampsia. Hromadnikova et al. (18) revealed miR-520a-5p downregulated during the first trimester of gestation for women destinated to develop FGR. Jiang et al. (19) found that urinary exosomes miR-21, miR-29a, and miR-590-3p can be used as potential biomarkers for the diagnosis of ICP, and these miRNAs could directly target and inhibit the expression of ICAM1. However, they selected differential miRNAs associated with cholestasis in urinary exosomes to validate according to the research on relevant literature. As far as we know, there are few studies on serum exosome miRNAs as non-invasive biomarker for ICP, and we constructed the differential expression lineage of ICP serum exosome miRNAs for the first times.

Our previous study demonstrated that a total of 19 differentially expressed miRNAs were founded; of them, 16 were found to be upregulated and three were found to be downregulated in serum samples obtained from pregnant women with ICP (9). In this study, we found a total of nine differentially expressed miRNAs in serum exosomes, the overall number of which was lower than that of expressed miRNAs in serum. At the same time, we found that among the differentially expressed miRNAs in serum exosomes, the proportion of downregulated miRNAs was significantly higher than that of upregulated miRNAs, which was contrary to the results of our previous studies on differentially expressed miRNAs in serum. In this study, GO enrichments and KEGG pathway analysis findings were consistent with the results of serum miRNA pathway analysis (9); lipid metabolism, apoptosis, oxidative stress, and the MAPK signaling pathway were found to be significantly altered *via* both methods. The hsa-miR-4271, hsa-miR-1275, and hsa-miR-6891-5p are closely related to these pathways and were therefore selected as targets for validation. At the same time, few studies were founded on the diagnostic value of ICP by hsa-miR-4271, hsa-miR-1275, and hsa-miR-6891-5p.

The hsa-miR-4271 targets the 3′UTR of constitutive androstane receptor (CAR). It has been mentioned in CAR expression, lipid metabolism, and Parkinson’s disease (20–22). Hu et al. (21) study shown that the level of miR-4271 in human hepatocellular carcinomas (HepG2) was more than 293T cell, which indicated that hsa-miR-4271 may play an important role in lipid metabolism in the liver. Wang et al. (22) founded that hsa-miR-4271 was negatively correlated with CAR expression and its downstream gene ATP-binding cassette subfamily B member 1 (ABCB1). ABCB1, known as lipid transporter, mediates the transfer of cellular PL and free cholesterol (FC) (23). In the target gene prediction, we found 10 target genes associated with hsa-miR-4271, and our bioinformatics analyses indicated that hsa-miR-4271 and related target genes may be participated in disordered lipid metabolism apparent in patients with ICP. The AUC of exosomes hsa-miR-4271 was 0.861. The non-error rate, sensitivity, and specificity of hsa-miR-4271 were 84.3%, 93.3%, and 71.4%, indicating that this exosome miRNA may has high sensitivity and accuracy for diagnosing ICP. However, the specificity of this exosome miRNA is not high, and it is necessary to combine with other two exosome miRNAs to improve its specificity.

Recent studies have shown that hsa-miR-1275 binds to the 3′UTR of IGF-1R and acts as oncogene to promote the occurrence and development of tumor. The biological function of hsa-miR-1275 was to promote the proliferation, invasion, and metastasis of tissue (24). Liu et al. (25) indicated that hsa-miR-1275 directly binds to the 3′UTR of IGF-1R induce migratory and proliferative behavior in squamous cell carcinoma of head and neck cell. Tong et al. (26) revealed that the inhibition of hsa-miR-1275 induced myocardial injury through downstream target gene HK2, leading to the decreasing concentration of pro-inflammatory factors TNF-α and IL-1 and the increasing concentration of anti-inflammatory factors IL-10. We found seven target genes associated with hsa-miR-1275, and our bioinformatics analyses provide clues that hsa-miR-1275 and related target genes may influence the migration, proliferation, and inflammation of trophocyte that induce the pathophysiology of ICP. The AUC of hsa-miR-1275 was 0.886, and the non-error rate, sensitivity, and specificity of hsa-miR-1275 were 87.0%, 96.7%, and 75.0%. The AUC and non-error rate of this exosome miRNA was the highest among the three exosome miRNAs, indicating that hsa-miR-1275 has high sensitivity and accuracy for diagnosing ICP.

The hsa-miR-6891-5p is encoded with intron 4 of the Human Leukocyte Antigen B (HLA-B) transcript, which is the most conserved intron among class I HLA genes (27). A recent study identified a conserved hsa-miR-6891-5p that involves lots of immunological processes by regulating the expression of nearly 200 transcripts (28). Zhang et al. (29) found that hsa-miR-6891-5p was significantly downregulated in the tissues of atypical meningiomas patients who were resistant to radiotherapy, and the differentially expressed hsa-miR-6891-5p was enriched mostly in the fatty acid metabolic pathways. Here, we found three target genes associated with hsa-miR-6891-5p. The hsa-miR-6891-5p and related target genes may be a component of the disordered lipid metabolism apparent in ICP. The AUC of hsa-miR-6891-5p was 0.838, and the non-error rate, sensitivity, and specificity of hsa-miR-6891-5p were 86.9%, 98.3%, and 72.9%, respectively. Although the AUC of this exosome miRNA was the lowest, its sensitivity was the highest among the three exosome miRNAs, and the accuracy was also at a high level, indicating that this exosome miRNA also has high sensitivity and accuracy for diagnosing ICP.

On the basis of the above studies, we found that the specificity of these three exosome miRNA alone have certain limitations for diagnosing ICP. Therefore, we conducted cross-validation analysis of these three exosome miRNAs; MLR analyses showed that the AUC of hsa-miR-4271 combined with hsa-miR-1275 reached the highest value (0.982), and the non-error rate, sensitivity, and specificity of hsa-miR-4271/1275 were 94.1%, 96.6%, and 90.5%. At the same time, we found that the AUC of exosomes hsa-miR-4271, hsa-miR-1275, and hsa-miR-6891-5p combined detection was 0.976, slightly lower than hsa-miR-4271/1275, but the non-error rate, sensitivity, and specificity was up to 95%, 96.6%, and 92.9%, which may afford more reliable ICP diagnosis than the individual levels did.

## Conclusions

As a result, we identified three differentially expressed miRNAs in the serum exsomes of patients with ICP and found that a combination of hsa-miR-4271, hsa-miR-1275, and hsa-miR-6891-5p may be as a new biomarker to improve the diagnosis and prognosis of ICP. However, this is a preliminary work, the limitation of our study is that we only explore differentially expressed miRNAs in serum exosomes, but the origin of this exosomes is not clear. In the following work, we will further explore the specific source of serum exosomes and explore the pathogenesis of ICP.

## Data Availability Statement

The original contributions presented in the study are included in the article/supplementary material. Further inquiries can be directed to the corresponding authors.

## Author Contributions

TZ: Conceptualization, methodology, funding acquisition, and supervision. LL: Software and project administration. RD: Writing—Original draft. NY: Writing—review and editing. JW: Writing—Original draft. SZ: Resources. TW: Investigation. GW: Validation. XS: Validation. JC: Formal analysis. YZ: Data curation. JW: Resources. TY: Formal analysis. MC: Data curation. All authors contributed to the article and approved the submitted version.

## Funding

This study was supported by the National Natural Science Foundation of China (grant no. 82171674 and 81671489), the Major Research Foundation of Jiangsu Science and Technology Department (grant no. BE2017628), Jiangsu Province Health Committee Scientific Research Project (grant no. M2021023), Wuxi City Key Medical Talent of the Project of Invigorating Health Care through Science, Technology and Education (grant no. Z201601), Wuxi City Health Committee top-notch talent (grant no. BJ2020077), Jiangsu Province Health Committee “Six One Project” Top Talent Project (grant no. LGY2020023), Wuxi City Health Committee Youth Project (grant no. Q202032 and Q202121), Wuxi City Health Committee Translational Medicine Project (grant no. ZH202109), and Wuxi City Science and technology development Fund project (grant no. Y20212035).

## Conflict of Interest

The authors declare that the research was conducted in the absence of any commercial or financial relationships that could be construed as a potential conflict of interest.

## Publisher’s Note

All claims expressed in this article are solely those of the authors and do not necessarily represent those of their affiliated organizations, or those of the publisher, the editors and the reviewers. Any product that may be evaluated in this article, or claim that may be made by its manufacturer, is not guaranteed or endorsed by the publisher.

## Abbreviations

ICP, intrahepatic cholestasis of pregnancy; TBA, total bile acid; qRT-PCR, quantitative reverse transcriptase polymerase chain reaction; UTR, untranslated region; mRNA, messenger RNA; nm, nanometer; HELLP, hemolysis, elevated liver enzymes and low platelet count; PBS, phosphate-buffered saline; TEM, transmission electron microscopy; NTA, nanoparticle tracking analysis; WB, Western blot; GO, gene ontology; BPs, biological processes; CC, cellular component; MF, molecular functional; KEGG, Kyoto Encyclopedia of Genes and Genomes; ROC, receiver operator characteristic; CIs, confidence intervals; AUCs, referencing areas under the curves; SEs, means ± standard errors; ALT, alanine transaminase; AST, aspartate transaminase; EDS, exosome-depleted supernatant; GDM, gestational diabetes mellitus; FGR, fetal growth restriction; CAR, constitutive androstane receptor; HepG2, Human hepatoellular carcinomas 2; ABCB1, ATP-binding cassette subfamily B member 1; PL, phospholipid; FC, free cholesterol.
